# Functional analysis of human cancer-associated genes and their association with the testes and epididymis

**DOI:** 10.3892/ol.2013.1450

**Published:** 2013-07-08

**Authors:** XIU-FENG HUA, XUE-BO WANG, FU-JUN LIU

**Affiliations:** 1 Department of Endocrinology, Yu-Huang-Ding Hospital/Qingdao University, Yantai, Shandong 264000, P.R. China; 2 Clinical Laboratory, Yu-Huang-Ding Hospital/Qingdao University, Yantai, Shandong 264000, P.R. China; 3 Central Laboratory, Yu-Huang-Ding Hospital/Qingdao University, Yantai, Shandong 264000, P.R. China

**Keywords:** cancer, expressed sequence tags, digital differential display, epididymis, bioinformatics

## Abstract

Human cancer-associated UniGene sets (NCBI GeneBank) provide a platform for identifying differentially-expressed genes in human cancers. The present study identified and characterized a set of human cancer-associated genes using the Digital Differential Display (DDD) and functional analysis tools. A total of 1,904 genes were differentially expressed in 15 cancer types, including genes that had been previously shown to be specific in certain human cancers. A total of 274 genes were uniquely expressed in certain cancer types, including 37 genes that were highly expressed in the human testes and epididymis. These genes mainly functioned as ribosomal proteins, enzymes, receptors, secretory proteins and cell adhesion molecules. The most common domains that were encoded by the cancer-associated genes were those of cytochrome P450 CYP2D6, serpin and apolipoprotein A-I. A further gene ontology (GO) enrichment analysis revealed seven major functional clusters, which corresponded to the enriched pathways involved in cancer. The present study provides a source of cancer-associated genes and their functions. The results provide new insights into cancer biology and the involvement of highly-expressed epididymal genes in cancer biomarkers.

## Introduction

The identification of cancer-associated genes is crucial for understanding the molecular mechanisms that are involved in tumorigenesis and tumor development. These genes may contribute to or drive cancer development by their involvement in the functions or pathways that allow cancer cells to evade growth control, metastasis or invasion (1).

In the past decade, a number of cancer-associated genes have been identified using rapidly evolving biotechnologies, particularly high-throughput techniques, including gene expression microarrays and proteomics (2). For example, known and novel breast cancer genes have been identified using microarray-based comparative genomic hybridization (3). Colorectal cancer-associated genes have also been identified using combined computational methods (4) and deep transcriptome sequencing (5). Certain genes are well-known biomarkers in particular cancers, including BRCA1 and BRCA2 in breast cancer (6), PSA in prostate cancer (7) and WFDC2 in ovarian cancer (8). The associated biological functions of these genes provide clues for the research into cancer biology. However, the few cancer biomarkers that have identified do not act as individual units to perform biological functions. Through the development of sequencing techniques, integrated omics profiles provide the opportunity to identify additional cancer genes. Therefore, it is necessary to understand the mechanisms underlying tumorigenesis at an integrative level.

Public resources, including the public (GenBank, UniGene, Swiss_Prot) and ontological [Gene Ontology,(GO) consortium and Kyoto Encyclopedia of Genes and Genomes, (KEGG)] databases, currently offer complementary information for cancer gene identification (9). Furthermore, the increasing omics studies of human cancer have produced useful datasets, resulting in the establishment of cancer-associated databases. The Cancer Genome Anatomy Project (CGAP) aims to determine the gene expression profiles of normal, precancerous and cancerous cells (http://cgap.nci.nih.gov). The Cancer Genome Project uses human genome sequencing in order to identify the genes that are involved in the development of human cancer (http://www.sanger.ac.uk/genetics/CGP). The Cancer Genome Atlas (TCGA) accelerates our understanding of the molecular basis of cancer (http://cancergenome.nih.gov). Other derived cancer databases are also publicly available.

Among these online resources, the UniGene database (NCBI GeneBank; http://www.ncbi.nlm.nih.gov/unigene) allows the use of Digital Differential Display (DDD, http://www.ncbi.nlm.nih.gov/UniGene/ddd.cgi) for the rapid identification of genes whose expressions are altered between tissue types. The UniGene database includes expressed sequences from diverse species-, organ- and disease-derived cDNA libraries. The availability of various human cancer libraries in the UniGene database provides a platform for the rapid identification of selectively-expressed cancer genes.

The present study compares the expressed sequence tags (ESTs) that were identified in 15 types of cancer using the DDD tool. The differentially-expressed genes were bioinformatically evaluated for potential biological functions and pathways, and their expressions were evaluated in the human epididymis. These genes may be involved in regulating the initiation, development and progression of cancer, and may provide potential targets or markers for cancer prognosis, diagnosis, prevention and treatment.

## Materials and methods

### DDD analysis

The DDD tool was used to screen the selectively-expressed genes in various cancer types using the UniGene database to compare the number of times that sequences from the libraries were assigned to a particular UniGene cluster. The selected 15 types of human cancer libraries were for adrenal, bladder, breast, cervical, colorectal, esophageal, germ cell, glioma, head and neck, kidney, liver, lung, ovarian, pancreatic tumor and prostate cancer. The statistically significant differences in the EST counts between the cancer groups were determined using the Fisher’s exact test. P<0.05 was considered to indicate a statistically significant difference.

### Gene ontological analysis

All selectively-expressed cancer genes were broadly classified into several catalogs according to the GO annotation (www.geneontology.com) and the functions reported in the literature.

### Enrichment bioinformatics analysis

The protein identifiers (IDs) were uploaded to the Database for Annotation, Visualization and Integrated Discovery (DAVID; http://david.abcc.ncifcrf.gov) and the enrichment analyses of the GO terms, including the biological process, molecular function and cellular component, were performed using the functional clustering annotation tools. The default options with a high classification stringency were used. Finally, the cluster names were extracted from the most biologically relevant GO term that was assigned to that cluster.

### Pathway analysis

Ingenuity pathway analysis v9.0 (IPA; www.ingenuity.com; Ingenuity Systems, Redwood City, CA, USA) was used to analyze the pathways and networks that the cancer-associated proteins were involved in. The following settings were used: Reference set, ingenuity knowledge base (genes only); network analysis, direct and indirect relationships; 35 molecules per network; and 25 networks used per analysis. For all species, tissues and cell lines were used for the analysis. The IPA used the Fisher’s exact test to determine the significant pathways linked to the input protein set compared with the whole ingenuity knowledge base.

### Further characteristics of cancer-associated genes compared with the published literature

Highly-expressed human testicular and epididymal genes from a previous study (10–12) were compared with the present data. The overlapping epididymal genes were further characterized using microarray annotation (http://humanet.scbit.org/index.jsp). Secretory proteins and cell surface proteins are considered to be promising biomarkers. All cancer-associated proteins were compared with the serum/plasma proteome (13) in order to select the secretory proteins, and were also compared with the cell surfaceome (14) to select the cell surface proteins. A GO annotation was used to further filter the results.

## Results

### Summary of selectively-expressed genes in human cancers

**Figure 1:**
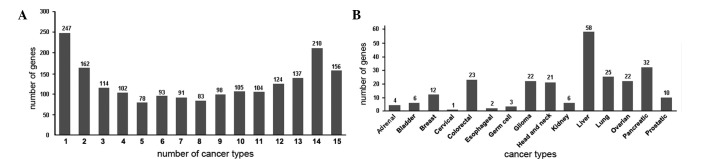
Distribution of 1,904 cancer-associated genes and 274 uniquely-expressed genes. (A) The numbers on the bars represent the number of genes; the x-axis represents the number of cancer types in which the number of genes in vertical coordinates were distributed. (B) Distribution of the 274 uniquely-expressed genes within the 15 types of cancer.

The statistical comparison of the ESTs among the various human cancer libraries was performed using the DDD tool. A total of 15 human cancer types and 208 libraries(1,196,901 ESTs) were selected (Table I). The genes that were significantly expressed in the various cancer types were termed the selectively-expressed cancer genes, and those that were uniquely expressed in one given cancer type were termed the uniquely-expressed cancer genes. A total of 1,904 selectively-expressed human cancer genes were identified in the 15 cancer types, of which 274 were uniquely-expressed cancer genes (Fig. 1).

### Bioinformatics analysis

**Figure 2:**
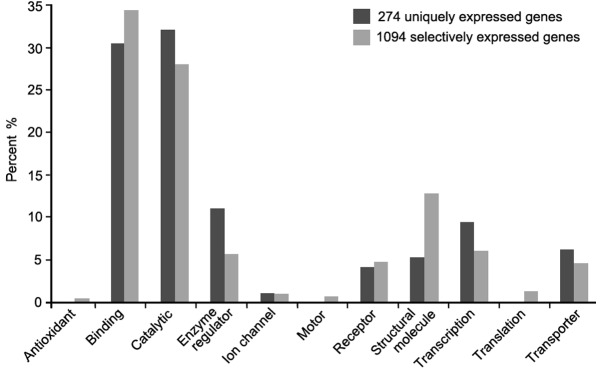
Broad functional classification of 1,094 cancer-associated genes and 274 uniquely-expressed genes.

The functional classification analysis showed that the majority of the selectively-expressed cancer genes were associated with binding (34%) and catalytic (27%) activity. Within the 274 uniquely-expressed cancer genes, non-related proteins were associated with antioxidant, motor or translation regulator activity functions (Fig. 2).

The domain analysis using the DAVID functional annotation tool from the PIR-superfamily classification system identified 13 and nine statistically significant domains in the selectively- and uniquely-expressed cancer genes, respectively. The common domains among the genes were cytochrome P450 CYP2D6 (PIRSF000045), serpin (PIRSF001630) and apolipoprotein A-I (PIRSF002367).

To map the major functional categories, all selectively-expressed cancer genes were grouped into several functional clusters using the functional annotation clustering tool (DAVID). The analysis revealed seven major functional clusters, including i) ribosome, ii) extracellular region, iii) cell adhesion, iv) acute-phase response, v) peptidase inhibitor activity, vi) regulation of cell death and vii) vasculature development.

An analysis of the pathway and the network was performed using IPA. A total of 21 statistically significant pathways were present in the selectively-expressed cancer genes (Table II) and 25 networks were generated. Certain functions were linked to more than three of the 25 networks and mainly included cancer development, cell death, the cell cycle, genetic disorder formation and cellular assembly and organization. The cancer category consists of numerous subcategories of which tumorigenesis is the largest.

### Comparison analysis

Certain genes that are highly-expressed in the testes or epididymis are promising markers for cancer (15,16). Of the 1,904 selectively-expressed genes, 52 were highly-expressed in the human testes and 30 in the epididymis. Of the 274 uniquely-expressed genes, 15 were highly-expressed in the human testis and 22 in the epididymis. A total of 360 secretory and 272 surface proteins were identified as potential biomarkers that corresponded with the selectively-expressed cancer genes.

### Characteristics of highly-expressed cancer genes in the human epididymis

Of the 274 highly-expressed genes in the human epdidymis, 22 exhibited distinct spatial and temporal expression patterns (Table III). According to the microarray data analysis (http://humanet.scbit.org/index.jsp), three genes were highly expressed in the caput epididiymis, seven in the corpus epididymis and three in the cauda epididymis. Seven genes showed higher expression levels in the aged males.

## Discussion

The identification of genomic markers that are associated with the progression of cancer is a key target in the field of cancer research. Genes that are selectively-expressed in cancer cells are promising targets for the development of new diagnostic and therapeutic markers (17). An ideal way to identify and characterize cancer-associated genes and their biological functions is to consolidate data from multiple comparable studies in order to perform an integrative analysis.

In the present study, cDNA libraries from 15 human cancer types were integrated for a statistical comparison. Through the utilization of the DDD tool, which screens statistically differentially-expressed genes between different libraries, a total of 1,904 selectively-expressed cancer genes were obtained, including 274 uniquely-expressed cancer genes within various cancer types. Certain genes are well-known to be specifically expressed in certain cancers, including WFDC2, which is uniquely-expressed in ovarian tumors and may be a promising marker in the diagnosis of ovarian carcinoma (18). A systematic bioinformatics analysis revealed significant biological functions that were enriched among these genes. These enriched functions or pathways will provide clues for further studies in cancer biology and may lead to the development of new diagnostic and therapeutic markers.

The functional characteristic analysis showed that the selectively-expressed genes mainly performed catalytic and binding activities. These genes shared common functional domains. Cytochrome P450 is a key enzyme domain in cancer formation and treatment (19), and the serpin domain contains cancer-related functions that are involved in tumorigenesis by regulating differentiation or inhibiting proteinases (20). Apolipoprotein A-I plays a significant role in the progression of ovarian cancer (21) and cholangiocarcinoma (22). These proteins are associated with the processes involved in cancer development.

The functional clustering analysis identified the main cellular components and biological process clusters that were enriched in the cancer genes. The ribosomal cluster, which is essential for protein synthesis, was the first to be identified in the analysis. It has also been reported that the ribosomal proteins contain certain extraribosomal functions, including those of apoptosis, DNA repair and RNA splicing and modification (23). Alterations in ribosome biogenesis is a cause of neoplastic transformation (24). Ribosomal protein expression has been shown to be differentially regulated in human colorectum carcinoma, in which ribosomal protein L7 has a neuroendocrine function (25). Notably, the selectively-expressed genes that corresponded to the ribosomal proteins were not specifically expressed in certain cancer types.

Cell adhesion molecules are known to play key roles in cancer progression through alterations of cell-cell and cell-extracellular matrix (ECM) adhesions, resulting in cancer cell migration, invasion and proliferation. In the present study, 24 cadherins, including 20 protocadherins and five integrins, were selectively expressed in the cancer cells. E-cadherin is expressed in normal epithelial tissues, but its suboptimal expression has been suggested to be associated with cancer invasion. E-cadherin immunohistochemistry is useful in diagnosing breast cancer (26). However, P-cadherin is frequently highly expressed in high-grade breast tumors (27). Further studies of these cell adhesion molecules may provide supporting information for the mechanisms of cancer cell interactions and metastatic activities.

The enriched functions of the cancer-associated genes are involved in various stages of cancer development and progression by participating in numerous cellular pathways or networks. Through IPA analysis, 21 significant pathways were identified, which corresponded to the enriched functional clusters.

Notably, 37 of the 274 uniquely-expressed genes were highly expressed in the human testes and epididymis. The cancer/testis genes that displayed a restricted expression in the testis and certain cancers are promising biomarkers. In total, >200 cancer/testis genes have been identified in the Cancer-Testis (CT) Antigens database (http://www.cta.lncc.br). The epididymis is subjected to rare tumors through several potential mechanisms. Certain selectively-expressed genes in the epididymis have spatial and temporal expression patterns for sperm maturation. The alternative expression levels of these genes in certain organs may act as an indicator of cancer development, including WFDC2 in ovarian cancer (28), ADAM29 and ADAM7 in melanoma (29) and Eppin in prostate cancer (30). However, to the best of our knowledge, no studies have reported the association between the highly-expressed epididymal genes and cancer. In the present study, it was hypothesized that certain genes that were strictly expressed in the epididymis may play specific roles in cancer biology.

In conclusion, the present study identified and characterized human cancer-associated genes and their biological functions. The genes that were differentially expressed in various cancer types mainly functioned as ribosomal proteins, enzymes, receptors, secretory proteins and cell adhesion molecules. The results provide a new insight into cancer biology and a new perspective into the involvement of highly-expressed epididymal genes in cancer biomarkers.

## Figures and Tables

**Table I:** Summary of libraries used in the present study.

Cancer type	Libraries	ESTs
Adrenal tumor	5	12384
Bladder carcinoma	2	8752
Breast tumor	16	88917
Cervical tumor	5	38138
Colorectal tumor	24	87951
Esophageal tumor	4	8672
Germ cell tumor	26	259108
Glioma	15	99668
Head and neck tumor	24	81627
Kidney tumor	13	68473
Liver tumor	24	102783
Lung tumor	15	139981
Ovarian tumor	15	61470
Pancreatic tumor	9	90266
Prostate cancer	11	48711

EST, expressed sequence tags.

**Table II:** Enriched pathways associated with selectively-expressed cancer genes.

Pathways^a^	EST count	Percentage	P-value
hsa03010: Ribosome	76	4.33	1.50×10^−53^
hsa04610: Complement and coagulation cascades	33	1.88	3.60×10^−11^
hsa04512: ECM-receptor interaction	28	1.59	9.53×10^−06^
hsa00010: Glycolysis/Gluconeogenesis	22	1.25	2.21×10^−05^
hsa00360: Phenylalanine metabolism	12	0.68	4.83×10^−05^
hsa04612: Antigen processing and presentation	23	1.31	1.31×10^−03^
hsa04510: Focal adhesion	43	2.45	2.60×10^−03^
hsa00982: Drug metabolism	18	1.02	3.06×10^−03^
hsa04115: p53 signaling pathway	19	1.08	3.55×10^−03^
hsa03320: PPAR signaling pathway	19	1.08	4.21×10^−03^
hsa05010: Alzheimer’s disease	34	1.94	1.16×10^−02^
hsa00350: Tyrosine metabolism	13	0.74	1.25×10^−02^
hsa00480: Glutathione metabolism	14	0.80	1.44×10^−02^
hsa00030: Pentose phosphate pathway	9	0.51	1.49×10^−02^
hsa00190: Oxidative phosphorylation	28	1.59	1.54×10^−02^
hsa05130: Pathogenic *Escherichia coli* infection	15	0.85	1.87×10^−02^
hsa03050: Proteasome	13	0.74	2.11×10^−02^
hsa04530: Tight junction	28	1.59	2.24×10^−02^
hsa05012: Parkinson’s disease	27	1.54	2.25×10^−02^
hsa05416: Viral myocarditis	17	0.97	2.74×10^−02^
hsa05215: Prostate cancer	20	1.14	2.94×10^−02^

a P<0.05.

EST, expressed sequence tags; ECM, extracellular matrix; PPAR, peroxisome proliferator-activated receptor.

**Table III:** Spatial and temporal expression of 22 genes that were highly-expressed in the human epididymis.

Gene symbol	Accession no.	Spatial expression			Temporal expression			Broad functions
	
		Caput	Corpus	Cauda	Newborn	Young	Aged	
ALDH3B2	NM_001031615	++	+++	++	+	++	+++	Aldehyde dehydrogenase family
ANO9	NM_001012302	+	+++	+	+	+++	++	Ion transport
C1QL1	NM_006688	+++	++	+	+	++	+	Locomotory behavior
CYB561	NM_182580	++	+++	+++	+	+++	++	Electron transport
ECM1	NM_004425	+	+++	++	+	++	+++	Signal transduction
EPN3	NM_017957	++	++	+	+	++	+++	lipid binding
GABRP	NM_014211	++	++	+	+++	+	++	Ion transport
GNAS	NM_000516	+++	+++	+++	++	+++	++	Signal transduction
GP1BB	NM_000407	++	+	+	+	++	+	Cell adhesion
GSN	NM_001127665	+	++	+++	+	+++	++	Calcium ion binding
NME2	NM_001018137	+++	++	+++	++	++	++	Transcription regulation
NPC2	NM_006432	+++	+++	+++	++	+++	+++	Lipid metabolism
NPFF	NM_003717	++	+++	+	+	++	+++	Receptor binding
PKM2	NM_002654	+++	+++	++	++	+++	+++	Glycolysis
PKP3	NM_007183	++	+++	+	+	++	++	Cell adhesion
RPL41	NM_021104	+++	+++	+++	+++	+++	+++	Ribonucleoprotein
RPS24	NM_001026	+++	++	++	++	+++	++	Ribonucleoprotein
SEMA4A	NM_022367	++	+++	+	+	++	+++	Receptor activity
TG	NM_003235	++	++	+	+	++	+++	Signal transduction
USH1G	NM_173477	++	+	+++	++	++	++	Actin cytoskeleton
WFDC2	NM_006103	+	+++	++	+	++	+++	Protease inhibitor
ZNF750	NM_024702	+++	++	+++	+	+++	++	Transcription regulation

The expression level of each gene is graded into three levels, strong (+++), moderate (++) and weak (+).
